# Automated Measurement of Cattle Dimensions Using Improved Keypoint Detection Combined with Unilateral Depth Imaging

**DOI:** 10.3390/ani14172453

**Published:** 2024-08-23

**Authors:** Cheng Peng, Shanshan Cao, Shujing Li, Tao Bai, Zengyuan Zhao, Wei Sun

**Affiliations:** 1 College of Computer and Information Engineering, Xinjiang Agricultural University, Urumqi 830052, China; p2392179899@163.com; 2 Agricultural Information Institute, Chinese Academy of Agricultural Sciences, Beijing 100080, China; caoshanshan@caas.cn; 3 Ministry of Education Engineering Research Centre for Intelligent Agriculture, Urumqi 830052, China; 4 Xinjiang Agricultural Informatization Engineering Technology Research Center, Urumqi 830052, China; 5 Hebei Tianhe Beef Cattle Farming Ltd., Shijiazhuang 050000, China; embryochina@163.com (S.L.); zhaozengyuanjsdx@163.com (Z.Z.)

**Keywords:** keypoint detection, beef cattle body measurements, precision livestock farming, non-contact measurement technology

## Abstract

**Simple Summary:**

In this study, we address the inefficiencies and animal welfare concerns associated with the traditional manual measurements of cattle dimensions by introducing a non-contact, automated measurement method. This method utilizes an improved keypoint detection model coupled with unilateral depth imaging technology. By improved the keypoint detection model, we have improved the model’s capability of processing critical cattle features. Subsequently, cattle body keypoints identified through conditional filtering of the depth image are optimized. Finally, these keypoints are integrated with various algorithms to compute the body size parameters of the cattle. In tests conducted on 23 beef cattle, the mean relative errors for body height, lumbar height, body length, and chest girth were 1.28%, 3.02%, 6.47%, and 4.43%, respectively. This research is of great significance for enhancing animal welfare and contributes to the sustainable development of modern livestock farming.

**Abstract:**

Traditional measurement methods often rely on manual operations, which are not only inefficient but also cause stress to cattle, affecting animal welfare. Currently, non-contact cattle dimension measurement usually involves the use of multi-view images combined with point cloud or 3D reconstruction technologies, which are costly and less flexible in actual farming environments. To address this, this study proposes an automated cattle dimension measurement method based on an improved keypoint detection model combined with unilateral depth imaging. Firstly, YOLOv8-Pose is selected as the keypoint detection model and SimSPPF replaces the original SPPF to optimize spatial pyramid pooling, reducing computational complexity. The CARAFE architecture, which enhances upsampling content-aware capabilities, is introduced at the neck. The improved YOLOv8-pose achieves a mAP of 94.4%, a 2% increase over the baseline model. Then, cattle keypoints are captured on RGB images and mapped to depth images, where keypoints are optimized using conditional filtering on the depth image. Finally, cattle dimension parameters are calculated using the cattle keypoints combined with Euclidean distance, the Moving Least Squares (MLS) method, Radial Basis Functions (RBFs), and Cubic B-Spline Interpolation (CB-SI). The average relative errors for the body height, lumbar height, body length, and chest girth of the 23 measured beef cattle were 1.28%, 3.02%, 6.47%, and 4.43%, respectively. The results show that the method proposed in this study has high accuracy and can provide a new approach to non-contact beef cattle dimension measurement.

## 1. Introduction

A United Nations report indicates that the global population will reach 9.7 billion by 2050 and peak at 11 billion by the end of this century [1]. The increase in population and changes in consumption patterns have heightened the demand for meat and dairy products, thereby spurring rapid development in the livestock sector. However, the expansion of livestock production has introduced a slew of environmental challenges. Sustainable livestock production and consumption not only pose challenges to the food system but also represent broader challenges for various stakeholders [2]. In modern livestock management, the element of cattle dimensions is a critical production metric. The size of cattle not only significantly influences their market value, genetic improvement [3], and management strategies [4] but also directly relates to their health status [5], meat quality [6], and reproductive performance [7].

In traditional livestock production, body weight and dimensions are typically measured using direct contact tools [8]. However, manual measurement methods require experienced workers, are time-consuming and labor-intensive, and the results are highly subjective, being easily influenced by external environments and experience. These measurements can also compromise animal welfare [9]. Non-contact sensor technology, as a relatively new and advanced method, provides a convenient means of real-time monitoring, aiding in the precision management of the livestock industry [10]. Non-contact methods for measuring cattle dimensions are crucial for the intensification and sustainable production of the livestock industry.

Currently, research on cattle body measurement mainly focuses on the following two technological approaches: two-dimensional image analysis and three-dimensional reconstruction techniques. Measurements using two-dimensional images generally involve capturing images of cattle with visible-light cameras. Subsequently, machine learning or deep learning technologies are applied to identify bovine features, which are then processed. Seo and others utilized a PC, a capture card, and two cameras to construct an image processing system. This system calculates body dimensions based on the relationship between image pixels and the actual dimensions of the cattle. Apart from the chest girth and pelvis height, the error in other body parameters is less than 5%. However, this method is sensitive to sunlight exposure and changes in the cattle’s posture, and the system structure is also relatively complex [11]. Tasdemir and others set up four Canon EOS400D cameras at the exit of the milking parlor to capture two images of the cattle from the side and the top. Using image processing software, they converted 2D image pixels into 3D spatial coordinates to calculate the cattle’s dimensions. Their research showed accuracies of 97.72% for wither height, 98% for hip height, 97.89% for body length, and 95.25% for hip width [12]. Viazzi and others noted that using two-dimensional images for body measurement in farm environments might not always be effective. Shadows and continuous changes in the background also make it challenging to capture image features accurately, and this can be well overcome by using depth cameras [13]. Spoliansky and others have shown that, although 2D sensors play an important role in body dimension detection, they lack the utilization of depth information provided by the third dimension compared to 3D sensors [14].

As the cost-effectiveness and practicality of consumer-grade depth sensors continues to improve, these technologies are increasingly penetrating precision agriculture and livestock management. Gao and colleagues used a ZED2I depth camera to capture images of corn plants and applied the modified YOLOv7-Pose to extract plant phenotypic parameters [15]. Rodríguez Alvarez and others utilized the Kinect v2 camera to capture images from the top of dairy cows and then employed the SqueezeNet model to automatically estimate the Body Condition Score (BCS) of the cows [16]. Miller and his team used a Time-of-Flight (ToF) camera to capture images of the back of cattle and employed artificial neural networks (ANNs) and Halcon software (MVTec Software GmbH, München, Germany) to predict weight based on 60 potential variables (including length, height, width, area, volume, and ratios), resulting in an R-squared score of 0.7 and a root mean square error (RMSE) of 42 kg [17].

For body dimension measurements, Ruchay utilized three Kinect v2 depth cameras to capture RGB-D data of cattle as they passed, including left, right, and top view depth images along with RGB images. Measurements such as body height, body diagonal length, and chest girth were calculated using 3D reconstruction and manual annotation of point cloud data [18]. Shuai designed a swine body dimension measurement system that uses three Kinect cameras positioned at different angles along the pen alley to collect data. The system then measures the swine’s dimensions after 3D reconstruction, achieving an average relative error of less than 5% for body length, height, width, and girth [19]. Huang and colleagues used a LiDAR sensor to capture raw data from Qinchuan cattle and employed techniques such as filter fusion, k-means clustering, RANSAC plane segmentation, FPFH feature detection, BRKD tree ICP registration, and GPT reconstruction. The cattle body dimensions were calculated using surface fitting and curve correction functions, resulting in an error close to 2 mm. However, this method requires different measurement systems to be built according to the body type of cattle at different ages, and the animals must remain as still as possible [20]. Le Cozler and others developed the Morpho3D automatic body measurement system using laser triangulation technology combined with five cameras and a laser projector to generate a detailed 3D point cloud of cattle bodies. Measurements are extracted from visually determined locations using Metrux2α^®^ software (3DOuest company, Lannion, France) [21]. Wang and his team obtained swine chest circumference slices through point cloud cross-sections and fitted curves to measure the perimeter for calculating the chest girth. The accuracy in detecting swine chest measurement locations was 96.5%, with an average relative error of 7.67% for chest girth [22]. Building on Wang’s work, Du combined the DeepLabCut toolkit to capture the key body-dimension points of pigs and cattle, mapping these points to the livestock point cloud to compute body dimensions. The average absolute error for cattle chest circumference was 10.76 cm, and it was 10.30 cm for swine [23]. Yang and colleagues captured about 55 images around dairy cows using a smartphone, employing Structure from Motion (SfM) photogrammetry, Random Sample Consensus (RANSAC), and Euclidean clustering to reconstruct the dairy cow point cloud. The calculations of key body dimensions showed that the relative errors for shoulder height, chest width, and chest girth mainly were below 5%, with most chest girth measurements being below 7.5% [24]. In summary, while point cloud or 3D reconstruction technologies can ensure the accuracy of livestock body measurements, these methods have certain limitations, as follows:

(1) Most related studies rely on specific data collection environments, lacking universality, which limits the widespread adoption of these technologies in various agricultural scenarios. (2) The deployment of multi-camera systems and body measurement equipment often involves significant costs. (3) These technologies frequently require manual marking of keypoints or rely on complex algorithms to extract features for calculating the specific dimensions of livestock, which is a laborious and error-prone process, especially in environments with limited computational and storage resources.

Given the many challenges faced by existing cattle size measurement technologies, such as low accuracy, high costs, and complex operations, this study aims to explore a new method combining keypoint detection models with single-side depth imaging. The scientific purpose of the research is to validate the applicability and accuracy of this technique in real farm environments, while the practical purpose is to simplify the measurement process, reduce costs, and improve efficiency. The method proposed in this study can effectively reduce costs and optimize the detection efficiency of cattle characteristic points while ensuring accuracy, which is of great significance in promoting the development of precision livestock farming.

## 2. Materials and Methods

### 2.1. Data Collection

The data collection site for this study is located at a professional beef cattle farm in Longhua County, Hebei Province, China. The subjects of the study are Simmental cattle who were raised in an environment that complies with national livestock welfare standards. The design of the livestock building takes into consideration ventilation, lighting, and spatial arrangement to ensure the health and welfare of the cattle. The bedding material is straw, which is regularly replaced to maintain dryness and cleanliness, preventing the occurrence of diseases. Microclimatic conditions such as temperature and humidity are regulated by an automated monitoring system to ensure they remain within the optimal range for cattle growth. The collection of image data was conducted in two phases, with the first being from 28 November 2023 to 14 December 2023 and the second being from 18 March 2024 to 29 March 2024, totaling 28 days. The selection of animals was based on the diversity of health conditions and ages to ensure the representativeness and scientific validity of the data. Furthermore, all the cattle included in the study came from the same herd, which consists of approximately 150 heads, to enhance the statistical power of the research.

To ensure high-quality data, the ZED2I depth camera was used to simultaneously capture side-view RGB and depth images of the cattle. The image data collection was facilitated using a laptop connected to the ZED2I depth camera, which was mounted on a tripod approximately 1 m high. The camera was positioned 1 to 5 m directly in front of the cattle’s abdomen, then set to record at 1080 p @30 fps. Video recording of the RGB and the corresponding depth images was synchronized to capture each cow as it naturally passed in front of the camera from appearance to disappearance from the lens. This method of collection ensured flexibility in image capturing while considering animal welfare, effectively capturing side images of the cattle in their natural state.

**Figure 1:**
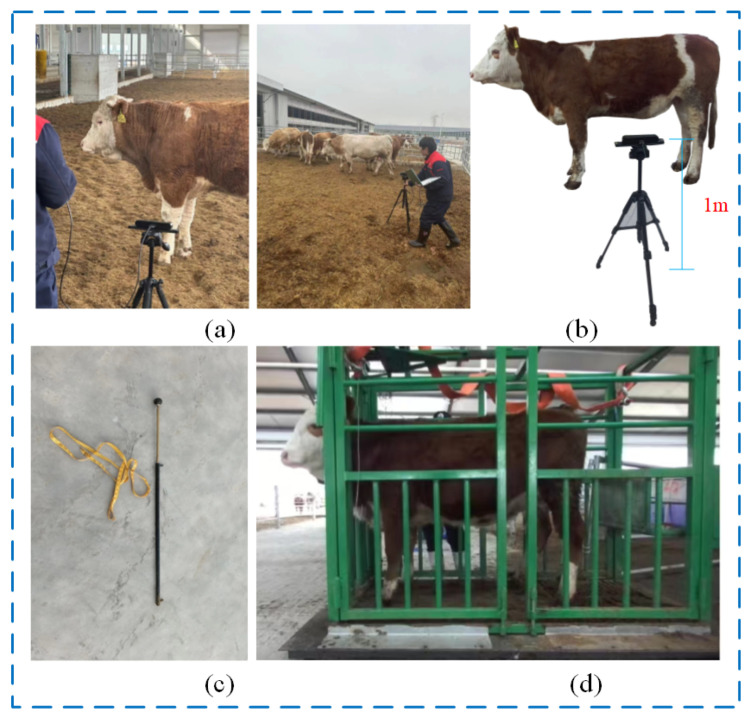
Data collection and body measurement scenes. (**a**) Image acquisition environment. (**b**) Image acquisition equipment. (**c**) Measurement equipment. (**d**) Body scale measurement environment.

To ensure data diversity and complexity, image acquisition was conducted at distances ranging from 1 to 5 m both inside and outside the barn during different time periods, ensuring effective capturing of cattle’s natural behaviors and postures under varying conditions. Actual body measurements of the cattle, including body height, lumbar height, chest girth, and body length, were precisely measured with the assistance of professional breeders using measuring sticks and tape measures. Ultimately, the study collected approximately 73 GB of video data of 95 cattle, including the actual measurement data for body height, body length, lumbar height, and chest girth for 23 cattle. The data collection and body measurement scenes are depicted in Figure 1.

### 2.2. Dataset Construction

**Figure 2:**
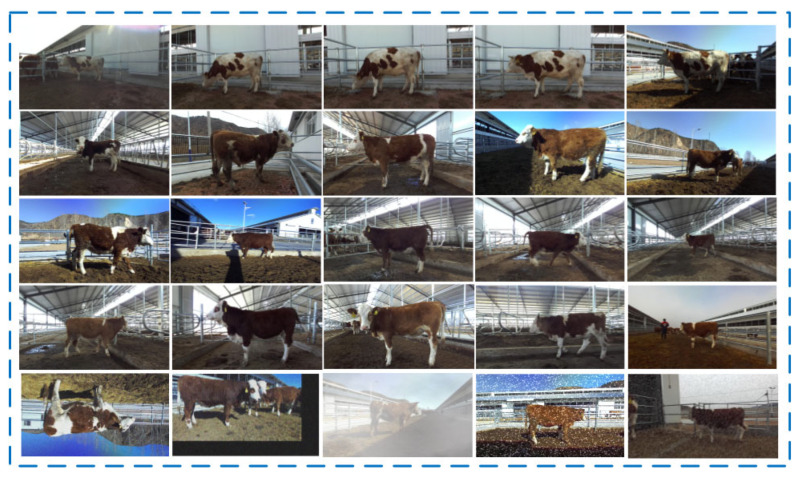
Dataset example graph.

We converted the 73 GB of video data from 95 cattle into RGB images and corresponding left-view depth images using the code provided by the ZED2i depth camera. From this, 2485 RGB images were selected, which include diverse data that vary in regard to distance, lighting, and posture. To ensure the generalizability and robustness of our model, we employed data augmentation techniques such as mirroring, rotating, translating, adding Gaussian and motion blur, and simulating different weather conditions to expand the dataset with an additional 500 augmented images. Ultimately, the 2985 images were split into a training set and a test set at a 7:3 ratio. Examples of the dataset are shown in Figure 2.

**Figure 3:**
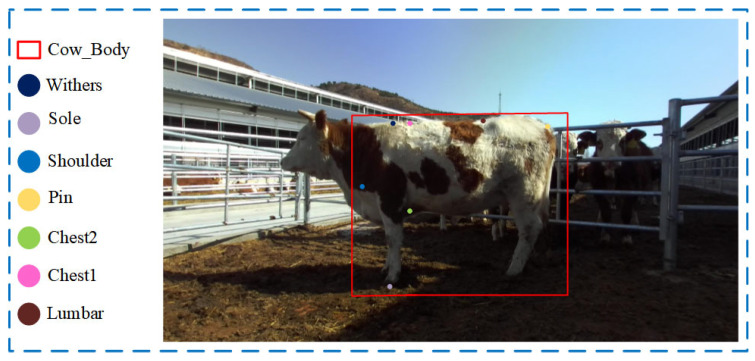
Data Annotation.

We used Labelme [25], a widely employed annotation tool in the field of computer vision, to mark keypoints on the bovine dataset. With assistance from professional livestock measurement experts, bounding boxes were used to label parts of the cattle body, named “Cow_Body”. Key anatomical landmarks such as the highest point of the withers, hoof, shoulder end, the external edge of the ischium, the rear edge of the withers, the vertical chest baseline, and the lumbar point were labeled, respectively, as Withers, Sole, Shoulder, Pin, Chest1, Chest2, and Lumbar. The data annotation is depicted in Figure 3.

### 2.3. Technical Route

**Figure 4:**
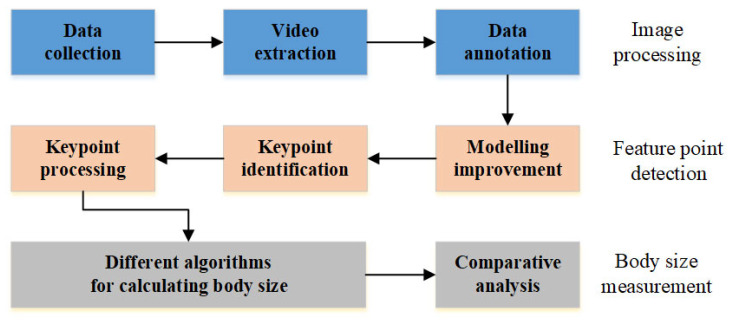
Technology Roadmap.

First, images of cattle, including single-sided depth images and RGB images, are acquired through data collection and video extraction processes. Then, the YOLOv8-pose model is enhanced to extract keypoints of the cattle based on the requirements of real-world scenarios. Subsequently, these points are refined and optimized on the depth images through conditional filtering. Finally, the cattle’s body height, lumbar height, body length, and chest girth are calculated using various algorithms. The technical roadmap is shown in Figure 4.

### 2.4. Improvements to Keypoint Detection Models

#### 2.4.1. Keypoint Detection Based on YOLOv8-Pose

Current 2D human pose estimation techniques are primarily divided into the following two approaches: top-down and bottom-up. The top-down approach first detects each human figure and then estimates the pose for each individually, which is suitable for complex scenes but computationally intensive. Conversely, the bottom-up approach identifies all keypoints first and then groups them, offering faster processing but potentially lower accuracy in densely populated scenes. YOLO-pose integrates the advantages of both methods based on the popular YOLO object detection framework. It accomplishes human detection and pose estimation in a single inference without the need for complex post-processing [26]. This method optimizes the keypoint similarities (OKSs) directly through end-to-end training, foregoing the conventional indirect loss functions based on heatmaps, significantly enhancing computational efficiency and accuracy. YOLO-pose exhibits excellent performance in the COCO dataset, demonstrating its efficacy and precision in practical applications, being particularly suitable for scenarios requiring high real-time performance and accuracy. Hence, we employ YOLOv8-pose for detecting cattle body keypoints.

#### 2.4.2. Content-Aware Restructuring Architecture: CARAFE

**Figure 5:**
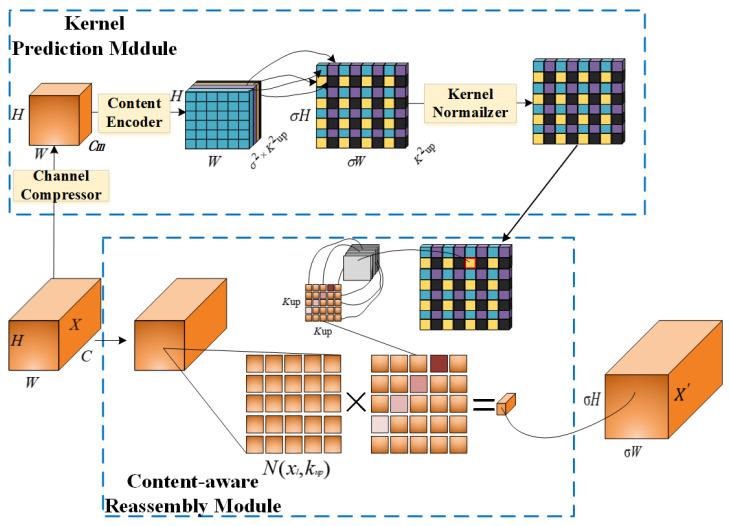
Overall architecture of CARAFE.

CARAFE (Content-Aware ReAssembly of FEatures) is an innovative feature upsampling technique that adaptively reassembles feature maps through a composite framework, as illustrated in Figure 5. This method primarily consists of the following two modules: the kernel prediction module and the content-aware reassembly module [27]. Initially, the kernel prediction module utilizes the input feature map X, generating an adaptive kernel through a channel compressor and content encoder. This adaptive kernel is dynamically produced by considering the content of the feature map and is subsequently standardized through a kernel normalization step. In the content-aware reassembly module, this adaptive kernel is used to spatially transform and reassemble the original feature map, producing the upsampled output feature map $X^{\prime }$. This method not only improves the accuracy of feature upsampling but also effectively maintains the spatial consistency and content dependency of features, which is particularly crucial for preserving the details and structure in the edge images of cattle bodies. The overall architecture of CARAFE is depicted in Figure 5.

#### 2.4.3. SimSPPF Network

**Figure 6:**
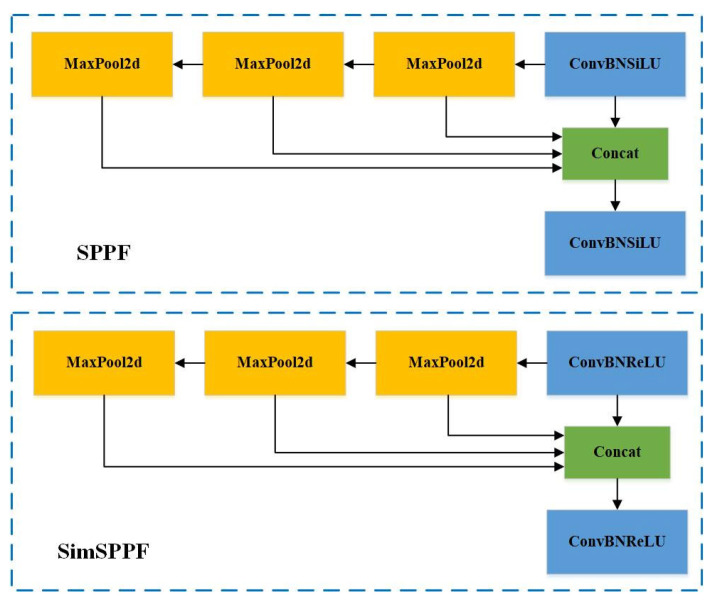
SimSPFF network structure diagram.

SimSPPF (Simplified Spatial Pyramid Pooling-Fast) is an optimized version of spatial pyramid pooling technology. The core of SPPF (Spatial Pyramid Pooling-Fast) is spatial pyramid pooling, a special pooling technique that performs pooling operations at multiple scales to capture multi-level features of images, enhancing the model’s robustness to scale variations. Introduced in YOLOv6 [28], SimSPPF simplifies this process compared to SPPF, reducing the complexity of convolution and pooling, and replaces the SiLU activation function with ReLU. ReLU features simple threshold operations, while SiLU involves more complex exponential computations. This change enhances performance and reduces the computational burden. By simplifying subsequent convolutions and activation functions, SimSPPF is better suited for resource-constrained environments, such as real-time applications on edge devices, providing a balanced solution between efficiency and performance. The network structure of SimSPPF is depicted in Figure 6.

#### 2.4.4. Improved YOLOv8-Pose Network Model Structure

In the original YOLOv8-pose network, the use of standard Upsample upsampling and SPPF (Spatial Pyramid Pooling-Fast) modules performs well in a variety of scenarios, yet there remains room for improvement in efficiency and accuracy, particularly for cattle keypoint detection tasks in complex environments. This is especially true for edge devices, where resources are limited and a more efficient and accurate model is necessary.

The traditional Upsample module implements upsampling through nearest neighbor or bilinear interpolation. Although simple, this method may lead to upsampling feature maps that need more content awareness and effectively utilize local features, impacting the accuracy of keypoint localization. The integration of the CARAFE (Content-Aware ReAssembly of Features) module allows for content-based adaptive upsampling. CARAFE enhances feature representation and keypoint detection accuracy by predicting an upsampling kernel at each location and reassembling features to generate finer feature maps. Additionally, while the original SPPF module increases the receptive field and captures multi-scale information, it does not maximize operational efficiency. Replacing SPPF with SimSPPF (Simplified Spatial Pyramid Pooling-Fast) reduces computational complexity and model parameters. SimSPPF lightens the model load with a simplified structure while still effectively capturing multi-scale information, making it suitable for use in environments with limited parameters and computational resources.

**Figure 7:**
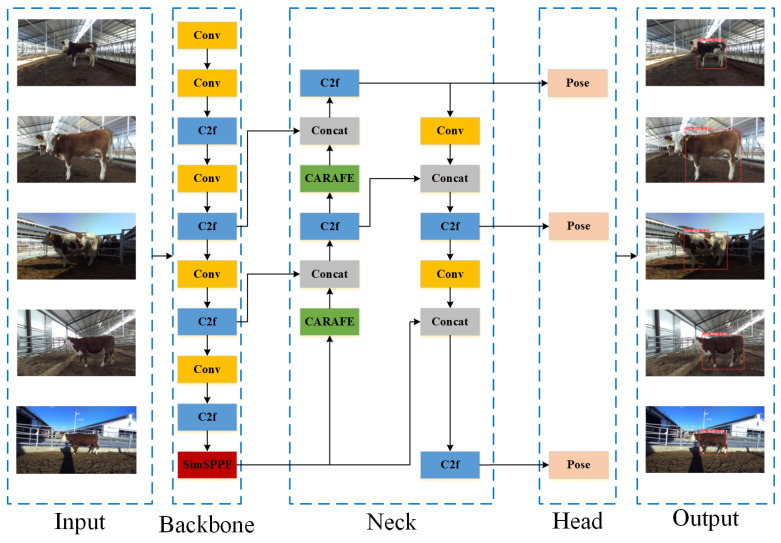
Improved YOLOv8-pose network structure diagram.

The improved YOLOv8-pose network, with optimizations in these two major modules, not only enhances the model’s ability to recognize cattle features in complex environments and improves keypoint detection accuracy but also optimizes computational efficiency, making it well suited for edge computing devices. With content-aware upsampling technology and an efficiency-optimized backbone network, the model ensures effective operation even on resource-constrained devices, providing support for real-time cattle monitoring and the keypoint measurement of body dimensions. The structure of the improved YOLOv8-pose network is shown in Figure 7.

### 2.5. Measuring Methods of Beef Cattle Body Size

#### 2.5.1. Keypoint Processing of Cow Body Depth Image

**Figure 8:**
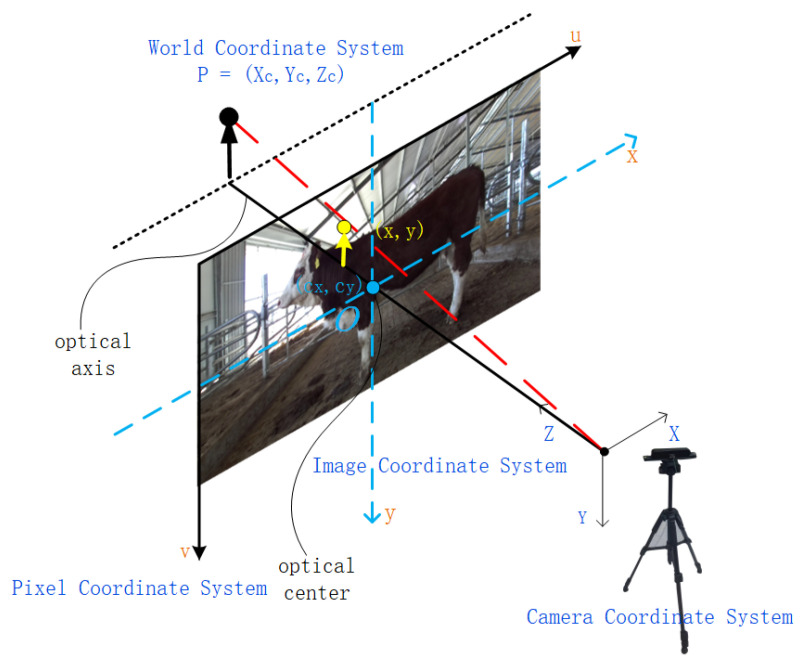
Schematic diagram of pixel coordinates to world coordinates conversion.

In this study, after obtaining the cattle body keypoints from RGB images using the improved keypoint detection algorithm, it is essential to ascertain their precise positions in three-dimensional space using information from the depth image. The accuracy of the keypoints identified on the depth image directly influences the effectiveness of cattle body dimension measurements. This process involves multiple steps to ensure optimal mapping and localization of keypoints on the depth image. Initially, we load the depth image stored in 16-bit unsigned integer format and convert it to a single-channel image. Based on multiple experiments, and in conjunction with our method of data collection using a depth camera aligned parallel to the abdomen of the cattle, we have identified the midpoint value between the shoulder and the ischial tuberosity of the cattle as a reliable reference for effective keypoint depth. The average depth of the midpoint and its surrounding points is calculated using neighborhood depth sampling, selecting the average of valid depths (0 to 7000 mm) within a 3 × 3 pixel window around the midpoint as the benchmark depth. This approach provides a stable measurement basis and reduces the impact of depth noise. Subsequently, within a set maximum radius, we locate the nearest valid depth where the difference from the midpoint’s average depth is less than 500 mm, enhancing the accuracy of keypoint depths. The keypoint localization algorithm is illustrated in Algorithm 1. Finally, by combining the image coordinates with the optimized depth values through inverse projection transformation and using the camera’s intrinsic matrix, the conversion to three-dimensional world coordinates is achieved, ensuring precise mapping from two-dimensional images to three-dimensional space. The specific conversion formula is shown in Equation (1), and a schematic diagram of the pixel coordinate transformation to world coordinates is presented in Figure 8.

(1) $$Z_{c}\left[\begin{array}{l} u\\ v\\ 1 \end{array}\right]=\left[\begin{array}{ccc} f_{x} & 0 & c_{x}\\ 0 & f_{y} & c_{y}\\ 0 & 0 & 1 \end{array}\right]\left[\begin{array}{c} X_{c}\\ Y_{c}\\ Z_{c} \end{array}\right]$$

where (u,v) are the pixel coordinates; (x,y) are the image coordinates; $\left(X_{c},Y_{c},Z_{c}\right)$ are the three-dimensional coordinates of the point in the camera coordinate system; $\left(c_{x},c_{y}\right)$ are the center coordinates of the image; $\left(f_{x},f_{y}\right)$ are the focal lengths of the camera.

The following Algorithm 1 is the pseudo-code for this part of the keypoint localization algorithm:
**Algorithm 1:** Depth Image Processing for Keypoint Localizationpath: input file path shoulder: coordinates of the shoulder endpoint pin: coordinates of the tuber ischium avg_depth: average depth value near the midpoint max_radius: maximum search radius K: internal parameter matrix coords: stores the converted world coordinates 1: **Function** LoadDepthImage(path): 2: image ← cv2.imread(path,cv2.IMREAD_UNCHANGED) 3: **if** image = None **then** 4:  exit(1) 5: **else** 6:  **return** image[:,:,0] 7: **Function** GetAverageDepth(image,shoulder,pin): 8: mid ← ((shoulder + pin)/2) 9: **return** mean depth at 3×3 gird around mid 10: **Function** FindNearestValidDepth(image,x,y,avg_depth,max_radius): 11: **for** dx ∈ [−max_radius,max_radius] **do** 12:  **for** dy ∈[−max_radius,max_radius] **do** 13:   depth ← image[y+dy][x+dx] 14:   **if** 0 < depth ≤ 7000 **and** |depth − avg_depth| ≤ 500 **then** 15:    **return** depth 16: **Function** GetWorldCoordinates(image,keypoints,K): 17: coords ← {} 18: **foreach** key,(x,y) ∈ keypoints **do** 19:  depth ← image[y][x] 20:  **if** depth **valid then** 21:   coords[key] ← K^−1^ ∙ [x,y,1] ∙ depth 22: **return** coords

#### 2.5.2. Body Height and Body Length Calculation

**Figure 9:**
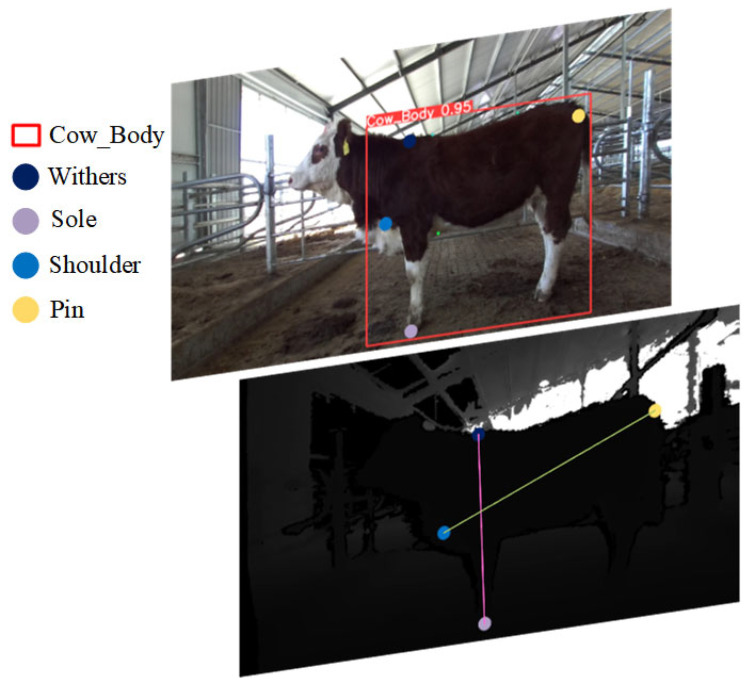
Schematic diagram of body height and body length calculation.

In the field of livestock and animal science, professionals commonly use measuring sticks and tape measures to measure the body height and body oblique length of cattle. The body height is measured by placing the measuring stick vertically on the ground and aligning it with the highest point of the withers of the cattle, reading the scale value. The body oblique length is measured using a tape measure, stretching it straight from the shoulder endpoint (Shoulder) to the outer edge of the tuber ischium (Pin) along the side of the cattle, recording the distance between these two keypoints. Mimicking the manual measurement method, we use the Euclidean distance formula to calculate the spatial length from the highest point of the withers (Withers) to the bottom of the hoof (Sole) to obtain the cattle’s body height. Similarly, the body oblique length calculation is derived from the Euclidean distance d between the shoulder endpoint (Shoulder) and the outer edge of the tuber ischium (Pin). The Euclidean distance formula is shown in Equation (2), and the calculations for body height and body oblique length are illustrated in Figure 9.

(2) $$d=\sqrt{{\left(X_{2}-X_{1}\right)}^{2}+{\left(Y_{2}-Y_{1}\right)}^{2}+{\left(Z_{2}-Z_{1}\right)}^{2}}$$

where $\left(X_{2},Y_{2},Z_{2}\right)$ and $\left(X_{1},Y_{1},Z_{1}\right)$ are the world coordinates of two points, respectively.

#### 2.5.3. Lumbar Height Calculation

**Figure 10:**
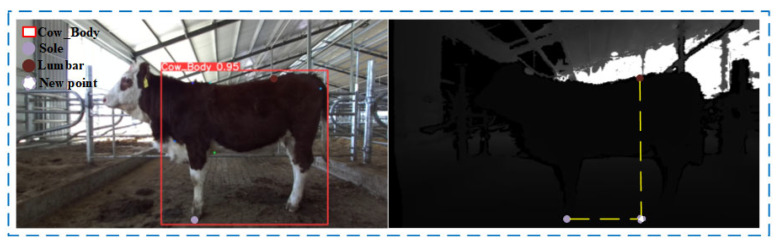
Schematic diagram of lumbar height calculation.

Professionals typically use a measuring stick to measure the lumbar of cattle manually. The operator places the measuring stick vertically on the ground, ensuring that its base is flush with the ground and the top end is snug against the cattle’s lumbar point. The height reading on the stick represents the lumbar height. In this study, we first identify the sole point (Sole) as a key marker for contact with the ground, with its depth value reflecting the distance from the depth camera to the ground. Subsequently, we combine the lateral coordinate of the lumbar point (Lumbar) with the longitudinal coordinate of the sole, and, using the depth value of the sole point (Sole), we construct a new three-dimensional point located on the ground plane directly beneath the lumbar point. Finally, the lumbar height is calculated using the Euclidean distance between the lumbar point (Lumbar) and this new three-dimensional point. The calculation of lumbar height is illustrated in Figure 10.

#### 2.5.4. Chest Girth Calculation

Professional measurers typically stand at the side of the cattle, wrapping a tape measure from the rear edge of the withers (Chest1) around the chest to the vertical point of the chest base (Chest2), ensuring the tape is horizontal and snug against the cow’s skin, and recording the measurement on the tape as the chest girth. Previous methods of measuring cattle chest girth often required the use of two or more depth cameras to create three-dimensional reconstructions or point clouds from the depth images obtained. These methods are generally costly, difficult to maintain, and slower in generating and processing point clouds compared to simple two-dimensional image processing, especially when dealing with large data volumes, which may affect real-time analysis and processing capabilities.

**Figure 11:**
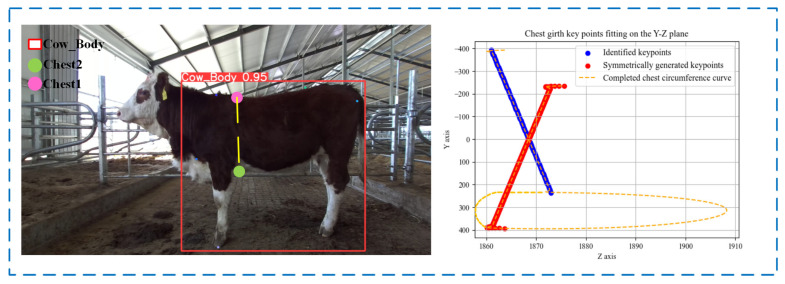
Chest circumference calculation curve.

This study proposes a method using depth imaging combined with machine learning techniques to estimate livestock chest girth. Initially, several intermediate points are generated between the established 3D space keypoints Chest1 and Chest2, forming a continuous curve segment in three dimensions. The upper half’s intermediate points are symmetrically transformed along the *Y*-axis to create mirror points on the other side of the chest girth. Next, to optimize the spatial positioning of these intermediate points, the Moving Least Squares (MLS) method [29] and Radial Basis Function (RBF) [30] are applied to adjust the set of points. This method not only considers the position of each point but also the distribution of nearby points to fine-tune each point’s coordinates, effectively addressing local anomalies caused by outlier points and ensuring the curve’s overall consistency. Through RBF processing, the coordinates of each interpolation point are recalculated, enhancing the overall structural stability of the point set while maintaining the continuity of the curve. After obtaining the data points curve for the upper half, the cubic B-spline interpolation method [31] is used to predict and complete the lower half of the chest girth curve. Finally, the distances between adjacent points on the loop are summed using the Euclidean distance formula to calculate the chest girth. The pseudo-code for chest girth calculation and its visualization are shown in Algorithm 2 and Figure 11.

The following Algorithm 2 is the pseudo-code for this part of chest girth calculation algorithm:
**Algorithm 2:** Calculate Chest Circumference from Depth ImageChest1: coordinates of the rear edge of the withers Chest2: coordinates of the vertical point of the chest base t: a linear space smooth_factor: smoothing factor rbf_x, rbf_y, and rbf_z: radial basis function interpolation objects t_new: newly generated time series tck: parameters of the strip interpolation x_fine, y_fine, and z_fine: interpolated x, y, and z coordinates 1: **Function** GenerateIntermediatePoints(world_coords,num_points): 2:  point1 ← world_coords[“Chest1”] 3:  point2 ← world_coords[“Chest2”] 4:  t ← np.linspace(0,1,num_points) 5:  **return** point1 + t[:,None] ∙ (point2 – point1) 6: **Function** MlsFitting(points,smooth_factor): 7:  x,y,z ← points[:,0],points[:,1],points[:,2] 8:  rbf_x ← Rbf(np.arange(len(x)),x,’multiquadric’,smooth = smooth_factor) 9:  rbf_y ← Rbf(np.arange(len(y)),y,’multiquadric’,smooth = smooth_factor) 10: rbf_z ← Rbf(np.arange(len(z)),z,’multiquadric’,smooth = smooth_factor) 11: t_new ← np.linspace(0,len(x) – 1,len(x) × 10) 12: **return** np.vstack(rbf_x(t_new),rbf_y(t_new),rbf_z(t_new)).T 13: **Function** CompleteEllipse(upper_points): 14: mirrored_points ← np.copy(upper_points) 15: mirrored_points[:,1] ← − mirrored_points[:,1] 16: all_points ← np.vstack(upper_points,mirrored_points) 17: (tck,u) ← splprep([all_points[:,0],all_points[:,1],all_points[:,2]],s = 0) 18: u_fine ← np.linspace(0,1,len(all_points) × 10) 19: (x_fine,y_fine,z_fine) ← splev(u_fine,tck) 20: **return** np.vstack(x_fine,y_fine,z_fine).T

### 2.6. Evaluation Indicators

#### 2.6.1. Keypoint Detection Model Evaluation Indicators

In this study, we utilize the Object Keypoint Similarity (OKS) to quantify the degree of match between the predicted keypoints and the true keypoints. The OKS is a widely used metric for human pose estimation tasks and is applicable for evaluating the performance of any type of keypoint detection, including animals. The formula for calculating the OKS is shown in Equation (3).

(3) $$OKS_{P}=\frac{\sum_{i}\;exp\left\{-d_{pi}^{2}/2S_{pi}^{2}\sigma_{i}^{2}\right\}\delta \left(v_{pi}>0\right)}{\sum_{i}\;\delta \left(v_{pi}>0\right)}$$

$$\delta =\left\{\begin{array}{l} =1\left(v_{pi}>0\right)\\ =0\left(v_{pi}\leq 0\right) \end{array}\right.$$

where $d_{pi}$ represents the Euclidean distance between the predicted position and the true position of the i keypoint. p and i represent the IDs of the target cow and keypoints, respectively. $S_{p}$ represents the object scale of p. $\sigma_{i}$ represents the normalization factor of the i keypoint. $v_{i}$ represents the visibility of the i keypoint, 0 means unmarked, 1 means marked but obscured, and 2 means marked and visible.

Therefore, we use the threshold defined by the OKS to calculate the Average Precision (AP). If the OKS is greater than 0.5, the result is classified as a true positive; if OKS is less than or equal to 0.5, the result is classified as a false positive. Thus, the mean AP calculated at an OKS value of 0.5 is used as the evaluation metric. The formula for calculating AP is shown in Equation (4), and the formula for mAP is shown in Equation (5).

(4) $$AP=\frac{\sum_{p}\;\delta \left(OKS_{p}>0.5\right)}{\sum_{p}\;1}$$

(5) $$mAP=\frac{\sum_{i=1}^{N}{AP}_{i}}{N}$$

Additionally, to ensure the efficiency and reliability of the model in various real-world application scenarios and to lay the foundation for practical deployment and application, we use Precision, Recall, GFLOPs, and Parameters as performance metrics to measure model detection accuracy, robustness, computational complexity, and resource consumption. Equation (6) provides the formula for calculating Precision and Equation (7) provides the formula for calculating Recall.

(6) $$Precision=\frac{TP}{\left(TP+TN\right)}$$

(7) $$Recall=\frac{TP}{\left(TP+FN\right)}$$

#### 2.6.2. Body Size Measurement Evaluation Index

We employ Mean Absolute Error (MAE) and Mean Relative Error (MRE) as metrics to evaluate the accuracy of body dimension measurements, which offer distinct advantages. Firstly, MAE, a fundamental regression metric, directly reflects the average deviation between predicted and actual values, quantifying the precision of the body measurement model and delivering a clear measure of error. This is crucial for assessing the algorithm’s applicability across various scenarios.

Secondly, MRE, by calculating the error as a proportion of the actual value, offers a relative scale for error assessment. This method of measuring relative error better adapts to the scale differences in body measurements of cattle of varying sizes, enhancing the universality and robustness of the error evaluation. When dealing with cattle of diverse body types or significant differences in body dimensions in particular, MRE effectively represents the model’s stability and consistency across different scales. Therefore, by integrating both MAE and MRE, we provide a dual verification that allows for a comprehensive assessment of the body dimension measurement model’s performance. The formulas for MAE and MRE are shown in Equations (8) and (9).

(8) $$\mathrm{MAE}\;=\frac{1}{n}\sum_{i=1}^{n}\;\left|{\hat{x}}_{i}-x_{i}\right|$$

(9) $$\;\;MRE\;=\frac{100\%}{n}\sum_{i=1}^{n}\;\left|\frac{{\hat{x}}_{i}-x_{i}}{x_{i}}\right|$$

## 3. Results and Analysis

### 3.1. Improved Results of YOLOv8-Pose

#### 3.1.1. Comparison of Keypoint Detection Using Different Algorithms

To enhance the accuracy of keypoint detection in cattle, we evaluated several models, including YOLOv5-Pose, YOLOv7-Pose, RTMpose[32], YOLOv8-Pose, and an improved version of YOLOv8-Pose. As indicated in Table 1, while YOLOv5-Pose and YOLOv7-Pose require relatively more parameters and computational resources, RTMpose has the lowest GFLOPs but suffers from lower mAP and a larger model size, which could compromise keypoint detection performance. YOLOv8-Pose exhibited the highest accuracy and performed well in terms of parameter count and model size. This confirms the practical applicability and superiority of the improvements made to the YOLOv8-Pose keypoint detection model.

#### 3.1.2. Model Performance Ablation Experiment

In this study, the YOLOv8-pose network model was employed, with enhancements such as the incorporation of SimSPPF in the backbone to optimize spatial pyramid pooling within the convolutional neural network and the integration of CARAFE in the neck to improve feature upsampling. A series of ablation studies were conducted to assess the impact of these modifications on the overall model performance. These experiments were designed to pinpoint the specific contributions of each component towards enhancing the model’s keypoint detection accuracy in complex environments.

As shown in Table 2, using YOLOv8-pose alone resulted in a Precision of 95.7%, a Recall of 87%, and a mAP of 92.4%, with the model size being 6.17 MB. The introduction of CARAFE increased the Precision to 96.3%, although the Recall was slightly decreased to 86.6%, while the mAP improved to 93.5% and the model size slightly increased to 6.47 MB, demonstrating the benefits of CARAFE-enabled upsampling technology. Furthermore, the sole use of SimSPPF, despite a slight decrease in Precision to 93.5%, saw an increase in Recall to 88.4% and an enhancement in mAP to 93.8%, with the model size remaining unchanged, indicating the efficacy of SimSPPF in optimizing feature extraction efficiency. Ultimately, the simultaneous incorporation of CARAFE and SimSPPF showcased optimal performance, with the highest Precision being 96.7% and the mAP reaching a peak of 94.4%, while the model size only marginally increased to 6.45 MB. These results confirm that meticulous integration and optimization of functionalities can significantly enhance the performance of deep learning models while controlling the model size to ensure efficiency.

#### 3.1.3. Improved Keypoint Detection Results

**Figure 12:**
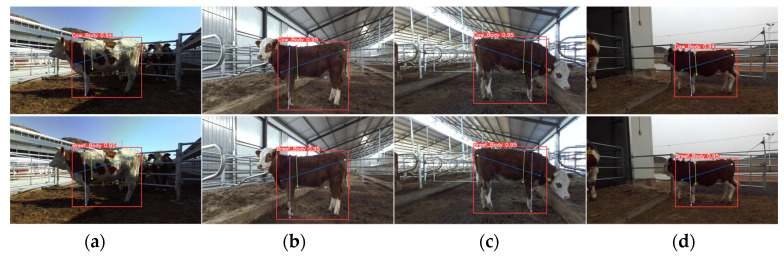
Keypoint prediction results. (**a**) In excellent lighting conditions; (**b**) with the cow standing; (**c**) with the cow bowing its head; (**d**) with the cow walking.

The enhanced YOLOv8-pose model was compared with the original model under various conditions. Figure 12a depicts a scenario with good outdoor lighting, Figure 12b shows the cattle standing, Figure 12c illustrates the cattle with their heads down, and Figure 12d captures the cattle walking. Due to the movement of the cattle or the influence of the surrounding environment, keypoints on the cattle’s body are prone to extend beyond the edges of the body. The improved YOLOv8-pose model is able to more accurately identify keypoints on the cattle’s edges in complex situations.

### 3.2. Body Size Measurement Results

#### 3.2.1. Normality Test

**Figure 13:**
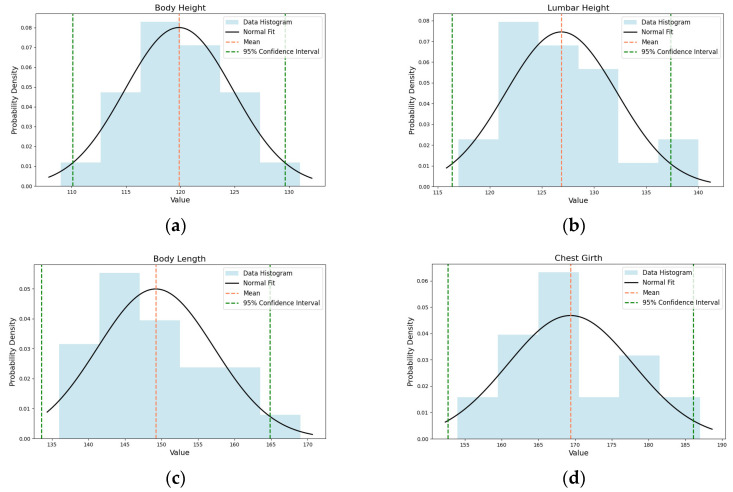
Normality test results: (**a**) indicates the results of body height; (**b**) indicates the results of lumbar height; (**c**) indicates the results of body length; (**d**) indicates the results of chest girth.

Normality testing ensures that the data conforms to the basic assumptions of many statistical analysis methods, which is an important step in assessing model accuracy, as standard error estimation methods typically assume data follow a normal distribution. By conducting Anderson–Darling tests, Shapiro–Wilk tests, and analyses of skewness and kurtosis [33,34] on manually measured cattle size parameters, we can accurately determine the distribution of small sample data. In Figure 13, the light blue histograms represent the frequency distribution of body size parameters, the black curve represents the normal distribution curve fitted based on the data’s mean and standard deviation, the orange dashed line represents the mean of the data, and the green dashed lines define the 95% confidence interval for the mean. From Table 3 and Figure 13, the following conclusions can be drawn: In the Shapiro–Wilk test, the statistics for the four parameters (body height, lumbar height, body length, and chest circumference) are close to 1, with *p*-values above the 0.05 significance level of 5%. In the Anderson–Darling test, the statistics for all parameters are below the critical value of 0.699 corresponding to the 5% significance level. Furthermore, the skewness and kurtosis of these body size parameters are close to 0, indicating that these parameters all exhibit characteristics of a normal distribution. These findings validate the normality of manually measured data, ensuring that the measurement results truly reflect the actual production situation, enhancing the practicality and universality of the study.

#### 3.2.2. Measurement Results and Analysis

**Figure 14:**
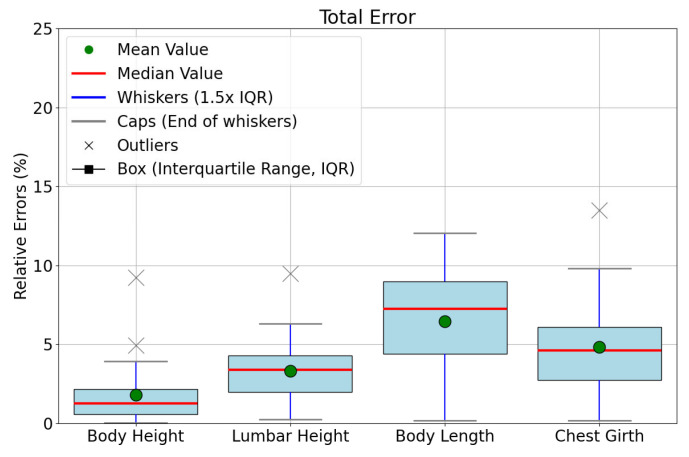
Body size measurement box plot results.

In this study, we measured the body dimensions of 23 cattle, including body height, lumbar height, body length, and chest girth. Using the improved YOLOv8-pose network model, we automatically detected keypoints on the cattle’s body and calculated the corresponding body dimensions based on these points. The measurement results are shown in Table 4, demonstrating the accuracy and consistency of this method across four types of body dimensions. Table 5 further summarizes the Mean Absolute Error (MAE), Mean Relative Error (MRE), and maximum relative error as follows: the average absolute error for Body height is 1.52 cm, with an average relative error of 1.28%, and a maximum relative error of 3.94%. The MAE for lumbar height is 3.83 cm, the MRE is 3.02%, and the maximum error is 6.30%. The MAE for body length is 9.77 cm, the MRE is 6.47%, and the maximum error is 12.03%, which may be influenced by the choice of measurement points or the posture of the cattle. The MAE for chest girth is 7.39 cm, the MRE is 4.43%, and the maximum error is 9.80%, reflecting the challenges in measuring complex body shapes. Additionally, Figure 14 further reveals the variability in error distribution across the four dimensions; the box plots for body height and lumbar height show very compact error distributions, indicating high consistency and reliability of measurements across the sample for these dimensions. The box plots for body length and chest girth reveal a relatively wider range of errors; the median and mean for body length are relatively far apart, possibly indicating potential challenges during measurement, such as chest girth measurements being affected by the breathing and muscle tension of the cattle, and body length is potentially affected by the natural standing posture of the animals.

Our study confirms that the method of automatic measurement of cattle body dimensions, based on improved keypoint detection combined with single-side depth images, is both practical and reliable. The method demonstrates significant accuracy and consistency, particularly in the measurements of body height, lumbar height, and chest girth, making it a valuable tool for real-world applications.

## 4. Discussion

In the discussion section of this paper, we meticulously explore the impact of various environmental factors on the accuracy of measuring cattle body dimensions. Specifically, this study systematically analyzes the specific effects of images with different noise levels, various measuring distances, and different cattle postures on cattle body dimension measurements. Discussing these factors is crucial for understanding and optimizing the application of automatic body measurement systems in real agricultural production environments. Additionally, we also thoroughly discuss the applications of the model.

### 4.1. Effects of Different Noises on the Measurement of Beef Cattle Body Dimensions

Different noises may affect the detection of keypoints on RGB images, thereby impacting the accuracy of body dimension measurements. To explore the impact of environmental noise, 51 randomly selected cattle images from the dataset were processed under simulated conditions of foggy, rainy, and snowy weather. In foggy conditions, image contrast and saturation are reduced, and slight Gaussian blur is added to simulate visual blurring and color fading, as fog can reduce image clarity. Rain simulation involves adding random noise and motion blur to mimic the dynamic impact of raindrops, as raindrops might be misidentified as part of the cattle. For snowy conditions, white noise, slight blur, and increased brightness threshold are used to simulate snow cover and bright winter environments, as the reflective properties of snowflakes can introduce optical noise.

**Figure 15:**
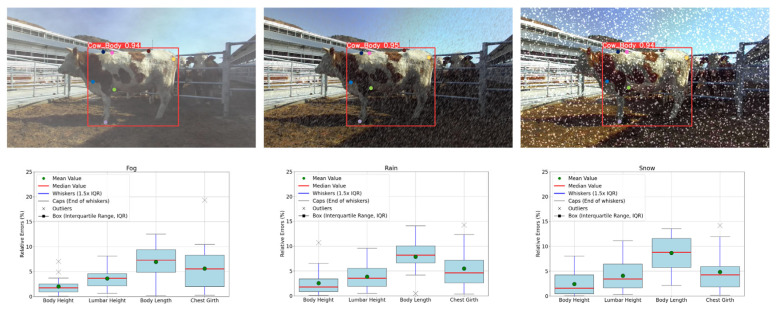
Results of keypoint detection and body size measurement under different noise conditions.

Specifically, in foggy noise environments, the Mean Absolute Error (MAE) for body height increased by 0.43 cm and the Mean Relative Error (MRE) increased by 0.36%; the MAE for lumbar height increased by 0.78 cm and the MRE increased by 0.58%; the MAE for body length increased by 0.67 cm and the MRE by 0.45%; and the MAE for chest girth increased by 0.96 cm and the MRE by 0.55%. In rainy noise environments, the MAE for body height increased by 1.09 cm and the MRE increased by 0.91%; the MAE for lumbar height increased by 1.11 cm and the MRE by 0.85%; the MAE for body length increased by 3.11 cm and the MRE by 2.09%; and the MAE for chest girth increased by 1.16 cm and the MRE increased by 0.67%. In snowy noise environments, the MAE for body height increased by 1.38 cm and the MRE by 0.96%; the MAE for lumbar height increased by 1.38 cm and the MRE by 1.07%; the MAE for body length increased by 3.25 cm and the MRE by 2.17%; and the MAE for chest girth slightly increased by 0.03 cm and the MRE increased by 0.02%. The results show that, even when cattle images are affected by blurring, noise, and other factors, the improved keypoint detection model maintains high recognition accuracy and robustness. Although MAE and MRE have increased, they are still within acceptable ranges, demonstrating the practicality of our automatic body dimension measurement system in complex environments. The effects of different noises on keypoint detection and body dimension measurement outcomes are illustrated in Figure 15.

### 4.2. Effects of Different Distances on the Measurement of Beef Cattle Body Size

**Figure 16:**
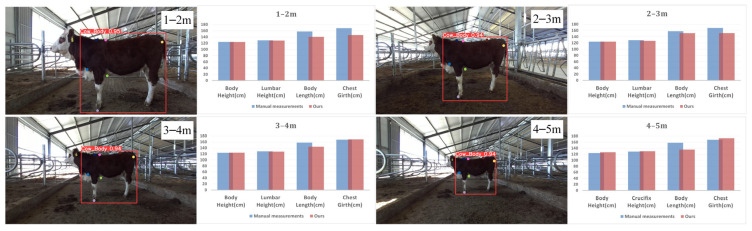
Keypoint detection and body measurement results at different distances.

We evaluated the accuracy of cattle body dimension measurements at different distances to explore the impact of distance on the measurements. We randomly selected cattle for study at distances of 1–2 m, 2–3 m, 3–4 m, and 4–5 m. We found that the impact of distance on measurement results showed clear differences. Specifically, the Mean Relative Error (MRE) within the 1–2 m distance range was 6.16%, while the MRE decreased to 3.89% in the 2–3 m range. Within the 3–4 m range, the error further reduced to 2.62%, but, in the 4–5 m range, the MRE increased to 5.07%. These results indicate a significant correlation between measurement accuracy and distance while also displaying that increasing distance does not always lead to increased error but rather shows a nonlinear trend, with 2–4 m being the optimal distance for the automatic body dimension measurement system. Moreover, even at longer distances (4–5 m), the resulting error remains within acceptable limits, validating the reliability and practicality of the measurement system used in this study for real-world applications. The impact of different distances on cattle body dimension measurements is shown in Figure 16.

### 4.3. Effects of Different Postures on the Measurement of Beef Cattle Body Size

**Figure 17:**
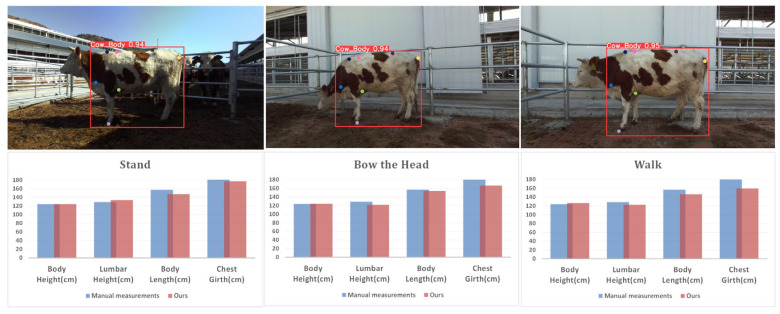
Keypoint detection and body measurement results for different postures.

Validating the robustness of the model under different cattle postures holds critical practical value for actual production. We randomly selected cattle to study their measurement accuracy in three common postures: upright standing, head-lowering, and walking. Results indicate that posture differences significantly impact measurement accuracy. The lowest mean relative error, at 3.83%, occurred in the upright standing posture, demonstrating high reliability in reflecting the natural body shape and dimensions of cattle. In the head-lowering posture, the stretching of the neck and back, along with adjustments in the shoulders and forelimbs, cause morphological changes in skeletal and muscle structures. This head-lowering action may cause displacement of key measurement points or obstruct the view, increasing the mean relative error to 4.62%. In the natural walking state, the muscular and skeletal structures of cattle exhibit significant dynamic adaptability. As cattle walk, their limbs move back and forth in a coordinated gait cycle, pulling muscle groups to contract and relax accordingly, maintaining balance and propulsion. This continuous dynamic change can cause real-time shifts in the positions of key measurement points, increasing measurement errors, reflected by a mean relative error of 7.04%. The impacts of different postures on cattle body dimension measurements are shown in Figure 17.

### 4.4. Model Application and Outlook

In the context of the rapid development of precision livestock farming [35], the importance of non-contact cattle body measurement techniques is increasingly prominent. The complexity of modern farming environments is primarily reflected in the variability of climatic conditions, diverse geographical environments, and varied farming methods. These factors make standardized management and monitoring extremely difficult. Although non-contact measurement methods using point clouds or 3D reconstruction ensure accuracy, they come with limitations such as high costs, complex algorithms, and dependence on specific measurement conditions.

**Figure 18:**
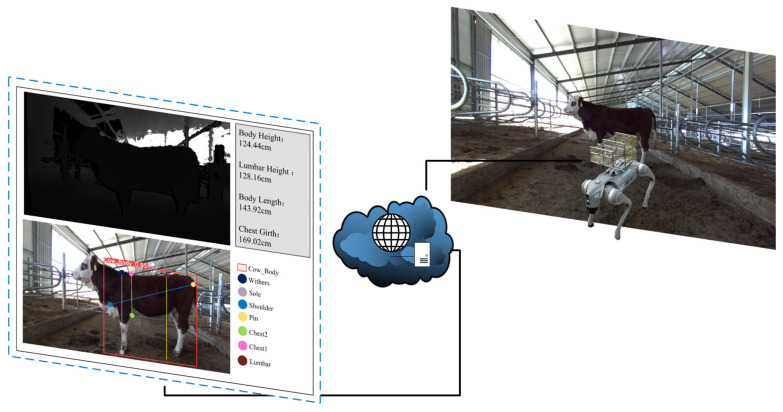
Non-contact body measurement system.

Against this backdrop, we have designed a new method for measuring cattle body dimensions using edge devices. These devices, such as robotic dogs, can autonomously navigate and operate in complex farm terrains without direct human–animal contact, significantly reducing labor intensity and enhancing operational flexibility. First, images captured by depth cameras mounted on robots are transmitted in real-time to a central server or cloud platform via wireless networks for processing. Then, the server runs an improved YOLOv8-pose model that can accurately identify keypoints on the cattle, such as the withers and chest base, in real-time. Finally, these keypoints are used by the server to calculate cattle body dimensions like body height, lumbar height, body length, and girth, which are then visually displayed for real-time viewing by veterinarians and farmers. The non-contact body measurement system is depicted in Figure 18.

In real application scenarios, where measurement of specific target cattle among multiple animals is required, we plan to integrate target detection [36], tracking technology [37] and individual identification techniques [38,39]. First, target detection and tracking technology can identify and monitor the dynamic positions of cattle in complex environments in real-time, which is crucial for continuous tracking of cattle’s natural behavior and social interactions. At the same time, individual identification technology ensures that each measurement is accurately attributed to the corresponding cattle. By combining these technologies, not only can efficient data collection be achieved, but the accuracy of the data and the precise identification of individuals can also be ensured.

## 5. Conclusions

This paper proposes an automatic cattle body measurement method tailored for practical scenarios, utilizing an improved keypoint detection technique based on the YOLOv8-pose model integrated with single-side depth images of cattle. The YOLOv8-pose model was selected, and its architecture was optimized by replacing the original SPPF with SimSPPF in the backbone for improved spatial pyramid pooling and incorporating CARAFE in the neck to replace the Upsample layer for upsampling. The improved YOLOv8-pose achieved an mAP of 94.4%, which is a 2% improvement over the baseline model. The improved model exhibits enhanced sensitivity to keypoints at the edges. Subsequently, cattle body keypoints from RGB images captured by the refined YOLOv8-pose were mapped onto depth images. These keypoints were then refined using conditional filtering and calculated using techniques such as Euclidean distance, Moving Least Squares (MLS), Radial Basis Function (RBF), and Cubic B-spline Interpolation (CB-SI) to determine the cattle’s body dimensions. Comparative analysis against the actual dimensions of 23 cattle revealed that the average absolute errors (MAE) for body height, lumbar height, body length, and chest girth were 1.52 cm, 3.83 cm, 9.77 cm, and 7.39 cm, respectively, with average relative errors (MRE) of 1.28%, 3.02%, 6.47%, and 4.43% and maximum relative errors of 3.94%, 6.30%, 12.03%, and 9.80%. These results indicate that the measurement method using single-side depth images of cattle is highly accurate and could reduce equipment costs, demonstrating potential for application in real farm environments and offering a new approach for non-contact cattle body measurement.

## Figures and Tables

**Table 1:** Keypoint detection results of different models.

Model	Parameters (M)	GFLOPs (G)	mAP (%)	Model-Size (MB)
YOLOV5-pose	7.12	16.7	89.7	54.83
YOLOV7-pose	9.61	20.1	91.2	19.6
RTMpose-t	3.34	0.3	91.0	50.3
YOLOv8-pose	3.10	8.4	92.4	6.17
Improved YOLOv8-pose	3.24	8.7	94.4	6.45

**Table 2:** Performance comparison of models under different improvements.

YOLOv8-Pose	CARAFE	SimSPPF	Precision (%)	Recall (%)	mAp (%)	Model-Size (MB)
√			95.7	87	92.4	6.17
√	√		96.3	86.6	93.5	6.47
√		√	93.5	88.4	93.8	6.17
√	√	√	96.7	87.4	94.4	6.45

**Table 3:** Normality test of artificial cattle body measurement.

Norm	Body Height	Lumbar Height	Body Length	Chest Girth
S-W Test	0.978	0.948	0.941	0.941
	0.885	0.275	0.196	0.197
A-D Test	0.261	0.491	0.608	0.598
Skewness	0.064	0.661	0.720	0.560
Kurtosis	−0.163	0.273	0.032	−0.403

**Table 4:** Body measurement results of 23 beef cattle.

ID	BH/cm		LH/cm		BL/cm		CG/cm	
1	126	124	126	129	146	157	187	187
2	132	131	133	140	154	169	180	187
3	128	127	128	136	145	163	188	181
4	127	127	124	137	148	162	181	180
5	124	122	126	128	146	146	171	166
6	124	124	128	129	144	158	169	168
7	111	109	113	117	127	138	167	154
8	123	123	129	130	153	153	158	167
9	119	118	120	123	132	146	176	169
10	121	118	127	127	150	156	162	173
11	118	114	118	120	128	145	151	159
12	113	114	116	121	134	145	171	167
13	129	118	124	129	136	147	170	166
14	124	121	126	130	137	151	182	172
15	115	114	115	123	147	146	165	160
16	123	119	123	126	133	141	156	164
17	118	114	118	122	139	144	170	167
18	132	123	132	127	145	153	153	177
19	124	119	124	124	134	148	186	178
20	118	118	118	124	134	141	147	163
21	123	122	123	127	139	147	151	166
22	118	117	118	123	140	146	149	163
23	123	121	123	126	139	150	154	162

**Table 5:** Measurement error analysis.

Index	Body Height	Lumbar Height	Body Length	Chest Girth
MAE(cm)	1.52	3.83	9.77	7.80
MRE(%)	1.28	3.02	6.57	4.65

## Data Availability

Data are available on request from the authors.
